# [Corrigendum] Survival outcomes in patients with T3‑4aN0M0 glottic laryngeal squamous cell carcinoma and evaluation of postoperative radiotherapy

**DOI:** 10.3892/ol.2024.14456

**Published:** 2024-05-15

**Authors:** Jian Zhou, Yu Heng, Yue Yang, Xiaoke Zhu, Liang Zhou, Hongli Gong, Chengzhi Xu, Lei Tao

Oncol Lett 24: 434, 2022; DOI: 10.3892/ol.2022.13554

Following the publication of this article, the authors have realized that an incorrect description of the treatment protocol was reported in the “*Postoperative procedures*” subsection of the “*Materials and methods*” on p. 2 concerning the three regimens on which platinum-based chemoradiotherapy (CRT) was based. The sentence commencing on line 15 of this section should have read as follows (changes are highlighted in bold): “[Platinum-based concurrent CRT was performed based on one of three regimens: i) cisplatin (DDP) regimen-DDP [**75 mg/m**^2^ (not “45–50 mg/m^2^/day”)] on days 1–3; ii) DDP combined with 5-fluorouracil (5-FU) (PF regimen)-DDP [**75 mg/m**^2^ (not “40 mg/m^2^/day”)] on days 1–3 and 5-FU [**650–700 mg/m**^2^ (not 750–800 mg/m^2^/day)] on **days 1–4** (not days 1–5); or iii) **carboplatin (CBP) regimen-CBP (AUC=5) on days 1–2** [not, as was reported, “carboplatin (CBP)-based regimen-CBP, calcium folinate, and tegafur (200, 300, or 1,000 mg/m^2^/day, respectively) on days 1–3 for 1–2 cycles].

The authors regret that this misinformation was reported in the text, and thank the Editor of *Oncology Letters* for allowing them the opportunity to publish this Corrigendum.

